# The Medical Code

**Published:** 1883-05

**Authors:** 


### THE MEDICAL CODE.

It seems to be the proper thing that each journal should express an opinion in respect to the recent ethical movement in the Empire State of the North.

It is too utterly true that there is secession—not rebellion—in New York. Who would believed it? Judge Bleckley, of Georgia, was right when he announced lately, that it is only the impossible that happens in our day.

What makes the matter worse, is that this break is per manent and irretrievable. There is no help for it. No· jurisdiction at the North can apply the principle of coercion, and we of the South, for many cogent reasons, have ever repudiated coercion.

Our New York brethren must go, set up for themselves and make the best of it, as this section so signally failed to do in 1865. And, mean time, we do not intend to lose our temper, call them ugly names, or charge them with hypocricy, selfishness and bad faith, much less rebellion.

No, no; we learned something in the experiences of the recent unpleasantness between the States. We do not think too highly of ourselves now, and we are more ready to do justice to every neighbor. But, time immemorial, the average Southerner has been a gentleman, whatever else he might be. He may be poor, he may be even largely lacking in that peculiar commodity called by the Boston exquisite, culchaw, and by the New York dude, the esthetic, but he is everywhere a gentleman.

He knows how to sympathise heartily with all who need sympathy; he is ready to aid the unfortunate and to make due allowance for the erring. He recognizes to its depths that great truth of humanity: "The heart knoweth his own bitterness and a stranger intermeddleth not with his joy."

The gentlemen leading and participating in the recent ethical departure of New York, are neither rebels, fools nor knaves. There are reasons and motives underlying the movement, unknown and unappreciated by the outside world—especially that part of which lies more than a thousand miles away from them.

The ox does not low over his fodder, but he sometimes lows lustily when the fodder is not there. And then there were heart-burnings and personal wrongs, together with strong, local pressure from environment incident to progressive changes that we can hardly appreciate, but which were serious things to them.

We think they have erred, but they are honest and true men for a' that, on whom we ask God's blessing. And when in after years we come dispassionately to write up the history of their secession, we shall not persist in calling it the Great New York Codal *Rebellion* of 1882.

---

Dr. D. N. Kinsman in the March number of the Cincinnati *Medical News*, says: "Too much stress has been placed upon the presence of the anatomical entity known as tubercle, and too little account has been taken of the condition tuberculosis, which must precede the tubercular eruption. This condition is as clearly connected with the subsequent eruption of tubercles as the stage of incubation of small pox is with the subsequent eruption."

Simple and obvious as this remark may appear, and however much it may smack of the mere contagium vivum theory for consumption, it is nevertheless eminently practical. To be forewarned is to be forearmed, and the steady mortality from this fell disease through the ages more than anything else illustrates the old adage: "An ounce of prevention is worth a pound of cure. The neglect of this principle augments the work of sextons everywhere and adds largely to the profits of many happy undertakers in Florida and Minnesota.

The writer says again: "H. Martin has shown that tubercle is to be known by its inoculability—and this capacity for inoculability extends indefinitely—the exposure of tubercle in a test-tube to a heat of from 60° to 85° F. for a short time developed in the material such virulence as to cause the death of guinea pigs in a few hours, with acute peritonitis and gangrene of the liver. While other guinea pigs inoculated with the fresh matter survived for weeks. Tuberculous matter exposed to the temperature of boiling water becomes inoctilous when inoculated."

Is the materies morbum of tuberculosis, then, parasitic? He proceeds: "Now if there is a contagion which can be transmitted from parent to child, we should expect ehe time would arrive, when, in cases where the diathesis is strongly marked from its existence in successive generations, the infection would pass beyond the fetus and effect a health matter during her pregnancy as occurs in syphilis. That consumption is contagious there can be no question. The number of experiments and the general harmony of their results admit of no reasonable doubt. Tubercle is inoculable and therefore contagious."

